# Quantification of relevance of quality of life assessment for patients with cognitive impairment: the suitability indices

**DOI:** 10.1186/1471-2377-14-78

**Published:** 2014-04-08

**Authors:** Karine Baumstarck, Mohamed Boucekine, Laurent Boyer, Valérie Aghababian, Nathalie Parola, Françoise Reuter, Anderson Loundou, Christophe Lançon, Jean Pelletier, Pascal Auquier

**Affiliations:** 1 EA3279 Self-perceived Health Assessment Research Unit and Department of Public Health, Aix-Marseille University, APHM, Marseille, France; 2 EA 3273 Psychology of Cognition, Language, and Emotion Research Centre, Aix-Marseille University, Marseille, France; 3 Department of Psychiatry, Sainte-Marguerite University Hospital, 13009 Marseille, France; 4 Departments of Neurology and CRMBM CNRS6612, Timone University Hospital, APHM, Marseille, France

**Keywords:** Cognitive impairment, Multiple sclerosis, Schizophrenia, Quality of life, Validity, Reliability, MusiQoL, SQoL

## Abstract

**Background:**

The extent to which MS patients with cognitive dysfunction can accurately self-report outcomes has been a crucial issue. The aim of this study was to quantify and compare the relevance of the quality of life (QoL) assessment between two populations with a high occurrence of cognitive dysfunction, specifically in individuals with multiple sclerosis (MS) and in individuals suffering from schizophrenia (SCZ).

**Methods:**

Design: A cross-sectional study was performed using the following inclusion criteria: MS and SCZ patients were diagnosed according to the McDonald criteria and DSM-IV criteria, respectively. Data on sociodemographic (age, gender, education level) and clinical (disease severity, disease duration) factors, QoL (disease-specific questionnaires, MusiQoL and SQoL) and cognitive performance (executive, memory, and attention functions) were collected. Non-impaired and impaired populations were defined according to the French norms. Psychometric properties were compared to those reported in reference populations, which were assessed in the respective validation studies. Suitability indices were provided used to quantitatively compare how the structures in the different populations matched with the initial structure of the questionnaires (reference populations).

**Results:**

One hundred and twenty-four MS patients and 113 SCZ patients were enrolled. Factor analysis was performed on the impaired populations and revealed that the questionnaire structure adequately matched the initial structure of the disease-specific QoL questionnaires. All of the suitability indices of construct and external validity in the non-impaired populations ranged from 70 to 100%.

**Conclusions:**

Our study suggested that cognitive dysfunction did not compromise the reliability or validity of the self-reported QoL questionnaires among subjects with cognitive dysfunction, such as MS and SCZ. Thus, this report may clarify the relevance of using self-reported QoL assessments in clinical practice.

## Background

The assessment of quality of life (QoL) has been considered increasingly important with regard to the evaluation of disease progression, treatment and the management of care provided to patients with chronic illness. The US Food and Drug Administration and the European Medicines Agency have provided detailed recommendations for QoL assessment [1,2]. Despite the acknowledged need to consider QoL issues, QoL assessment remains under-utilized in clinical practice [3,4].

QoL is commonly assessed using self-reported questionnaires. The extent to which patients with cognitive dysfunction can accurately self-report their QoL has been a crucial issue that has only been partially examined [5]. Few studies have investigated this issue in specific populations, such as in patients with multiple sclerosis (MS) [6,7], patients with serious chronic mental illnesses [8,9], in elderly populations [10,11], and in patients with traumatic brain injury [12]. These studies have produced conflicting results, where some studies have demonstrated that the cognitively impaired are unable [13,14] or able [6,7,9] to produce valid and reliable QoL measures. However, these studies provided restricted data regarding validity and reliability and did not report whether the structure of the questionnaire described in the impaired populations was well adapted when the QoL measure was used in these individuals. More recent studies have reported strong arguments in support of the conclusion that cognitively impaired patients can reliably and consistently respond to disease-specific questionnaires, where such studies used an interesting approach based on quantification of the relevance of the QoL assessment [15-17]. This quantification relied on suitability indices based on decision rules. These rules defined expected psychometric properties according to the appropriate standards [18,19] and the properties reported in the reference population in the initial validation publication of the QoL questionnaire. This approach enables the determination of the relevance of QoL measures in different populations independent of the questionnaire used.

In this study, we compared the relevance of the QoL assessment between 2 populations of subjects with cognitive dysfunction to determine if the nature of the disease influenced the individual’s ability to accurately report his or her life experience. Cognitive deficits occur in a large proportion of patients with MS [20,21] as well as in patients suffering from schizophrenia (SCZ) [22,23]. In these 2 populations, similar neuroimaging abnormalities were reported [24,25] and all cognitive domains, such as executive functions, memory, and attention/concentration are affected. The aim of this study was to explore the validity of disease-specific QoL questionnaires in patients with impaired cognitive function and to compare these findings between MS and SCZ patients.

## Methods

### Study design and participants

This study utilized a cross-sectional design. The inclusion criteria for MS patients included the following: an MS diagnosis according to the McDonald criteria [26], any subtype of MS, no neurological disease (other than MS), and no history of severe mental illness (except depression disorders). The inclusion criteria for SCZ patients included the following: age, diagnosis of SCZ according to the Diagnostic and Statistical Manual of Mental Disorders, 4th ed. (DSM-IV) criteria [27], and no neurological disease. All of the patients were over 18 years old, outpatients, in a stable disease phase (no relapse during the last 3 months), and native French speakers. MS patients were recruited from the neurology department of a public French academic teaching hospital (Marseille, France) and SCZ patients were recruited from two psychiatric hospitals (Marseille and Toulon, France). The French Ethics Committee approved this study (Comité Consultatif de Protection des Personnes dans la Recherche Biomédicale, Marseille, France). Informed consent was obtained from all subjects.

### Data collection

The collected data included sociodemographic information, clinical characteristics, QoL and cognitive assessments. The sociodemographic (age, gender, and education level) and clinical (disease duration) data were recorded for each patient. Disease severity was assessed using the Expanded Disability Status Scale (EDSS) [28] and the Positive and Negative Syndrome Scale (PANSS) for MS and SCZ patients [29], respectively.

QoL was assessed using the French versions of disease-specific questionnaires: the MusiQoL for MS patients and SQoL for SCZ patients. The MusiQoL is a well-validated MS-specific questionnaire [30] consisting of 31 items that describes nine dimensions and provides a global index score: activity of daily living (ADL), psychological well-being (PWB), symptoms (SPT), relationships with friends (RFr), relationships with family (RFa), relationships with the health care system (RHCS), sentimental and sexual life (SSL), coping (COP), and rejection (REJ). The SQoL is a self-administered QoL questionnaire designed for people with schizophrenia [31,32]. It consists of 18 items that describes 8 dimensions (psychological well-being (PsW), self-esteem (SE), family relationships (RFa), relationships with friends (RFr), resilience (RE), physical well-being (PhW), autonomy (AU) and sentimental life (SL)) and provides a total score (index). For the two questionnaires, the dimension and index scores range from 0, which indicates the lowest QoL, to 100, which indicates the highest QoL.

### Cognition assessment

For both patient groups, cognitive assessment included executive function, memory, and attention performances. The executive function performance was assessed using both the French versions of the Stroop color-word test [33] and the Trail Making test (TMT) [34]. Memory performance was assessed using the long-short-delay tests of the French version of Grober and Buschke [35] in MS patients, and the long-short-delay subscales of the French version of the Wechsler Memory Scale [36] in SCZ patients. Attention performance was evaluated using the attention/concentration subscale of the French version of the Wechsler Memory Scale [36]. The same trained psychologist administered the tests in a standardized manner and the same instructions were given to the subjects prior to each test. Applying French normative values according to age, gender, and educational level (except for the Wechsler Memory Scale), the patients were divided into the following categories of cognitive function: 1) executive function: non-impaired (normal Stroop test and normal TMT) and impaired (other cases) [37]; 2) memory function: non-impaired (normal short-delay memory and normal long-delay memory) and impaired (other cases) [35,36]; 3) attention function: non-impaired (normal attention/concentration subscale) and impaired (other cases) [36]. These categorizations enabled the characterization of each patient as a no or low-impaired (no impairment or one impaired function) and highly-impaired global function (2 or 3 impaired functions).

### Statistical analysis

Statistical analyses were performed on the 4 populations defined above, i.e., no/low-impaired and highly-impaired groups for the 2 diseases, using the same procedure reported in the initial validation publications (reference populations) for MusiQoL [30] and SQoL [32]. For each group, the psychometric properties were compared to those reported in the reference population as assessed in the validation study of each questionnaire [30,32]. The structures of the MusiQoL/SQoL were examined in the no/low-impaired and highly-impaired populations using factor analysis to determine how these structures matched with the initial structure of the questionnaire using principal component factor analyses with a varimax rotation [18,19]. For each population, the proportion of factors identified from the initial factor structure of the MusiQoL/SQoL and the proportion of items projected to their initial dimension were obtained.

The multidimensional structure (construct validity) was verified using the multi-trait/multi-item analysis program [38]. The internal structural validity was assessed by investigating item-dimension correlations. The item internal consistency (IIC) was calculated by correlating each item with its scale, and the item discriminant validity (IDV) was assessed by determining the extent to which the items correlated with the dimension that they were hypothesized to represent compared to the correlations with other dimensions. Floor and ceiling effects were determined to assess the homogeneous repartition of the response distribution. For each dimension, the internal consistency reliability was evaluated using Cronbach’s alpha coefficient [39].

The unidimensionality of each dimension was explored by computing the item goodness-of-fit statistics (INFIT), which was obtained from Rasch analyses [40]. The INFIT values ranged from 0.7 to 1.2 to ensure that all of the scale items measured the same concept.

To explore external validity, Spearman’s correlation coefficients were used to investigate the relationships between the MusiQoL and SF36 dimensions in each group, and the associations between the MusiQoL dimension scores and sociodemographic and clinical features were reported similar to the validation study. For qualitative variables, the mean dimension scores of the MusiQoL were compared across patient groups (e.g., gender, educational level, marital status, and occupational status) using one-way analysis of variance. Quantitative variables (e.g., age, EDSS score, and MS duration) were analyzed using Spearman’s correlation coefficients. Acceptability was assessed by calculating the percentage of missing data per dimension. Data analyses were performed using SPSS 11.0, MAP-R, LISREL and WINSTEP software.

To quantify the manner in which each of the 4 structures matched with the initial structure (2 reference structures), suitability indices were calculated as previously described [15]. Decision rules, which were established by experts in QoL, were used to define satisfactory properties according to appropriate standards [18,19]. The means of the different proportions were calculated to produce a suitability index of the ‘construct validity’ and a suitability index of the ‘external validity’.

## Results

One hundred and twenty-four consecutive MS patients and 113 patients with schizophrenia were enrolled in this study. The sociodemographic and clinical features are listed in Table 1. At the timepoint of performing QoL assessment, 118 (95%) MS patients were taking disease-modifying drugs and 109 (96%) patients with schizophrenia were taking atypical antipsychotics (average dose of chlorpromazine of approximately 570 mg). According to the French normative values and to the definition of global cognitive status, 50 MS patients were categorized as cognitively no/low-impaired and 49 patients were categorized as cognitively highly-impaired. In addition, 40 SCZ patients were categorized as cognitively no/low-impaired and 67 patients were categorized as cognitively highly-impaired.

**Table 1:** Sample characteristics

		**MS**		**SCZ**
**Sociodemographic characteristics**		**N = 124**		**N = 113**
Sex ratio (men:women)		53:71	Sex ratio	79:34
Age in years: M ± SD		45.05 ± 10.80	Age	38.60 ± 10.80
Educational level	<12 years	65 (52.8%)	<12 years	62 (54.9%)
	≥ 12 years	58 (47.2%)	≥ 12 years	51 (45.1%)
**Clinical characteristics**				
Disease subtypes	Relapsing remitting	36 (29.0%)	Paranoid schizophrenia	60 (53.1%)
	Primary progressive	20 (16.3%)		
	Secondary progressive	61 (49.6%)		
	Clinically isolated syndrome	7 (5.7%)		
Disease duration in years: median [range]		10 [0–31]		22 [19-27]
Severity: median [range]	EDSS	4.75 [1-8]	PANSS	61 [51–75]

MD ± SD mean ± standard deviation.

MS multiple sclerosis, SCZ schizophrenia.

EDSS Expanded Disability Status Scale.

PANSS Positive and Negative Syndrome Scale.

### Construct validity

In the MS sample population, seven of the 9 initial factors were identified in the no/low-impaired group (REJ and SSL dimensions were not clearly identified), and 8 initial factors were found in the highly-impaired group (COP dimension was not detected). Twenty-seven and 29 of the 31 items contributed to the initial dimension in the no/low-impaired and highly-impaired groups, respectively.

The proportion of dimensions with IIC, which was greater than 0.2 compared to the reference population, was higher in the highly-impaired population compared to the no/low-impaired population. The proportion of dimensions with IDV, which was not greater than 0.2 compared to the reference population, was moderately satisfactory in both populations. The correlation for each item with its contributive dimension (IIC) was higher compared to the other dimensions (IDV) for 6 and 7 dimensions in the no/low- and highly-impaired populations, respectively. Cronbach’s alpha coefficients were satisfactory for 6 of the 9 dimensions in the no/low-impaired group. In addition, both populations showed satisfactory INFIT statistics. The floor effects were less than 10% compared to the reference population for 8 and 4 dimensions in the no/low-impaired and highly-impaired groups, respectively. The proportion of dimensions with a ceiling effect exceeding 10% compared to the reference population was higher in the highly-impaired population compared to the no/low-impaired population. The suitability indices of the construct validity were 82% and 78% in the highly-impaired and no/low-impaired populations, respectively.

In the SCZ sample, the 8-factor structure of the SQoL was clearly detected in the no/low and highly-impaired groups. Sixteen of the 18 items contributed to the initial dimension in the no/low-impaired and highly-impaired groups, respectively. The proportion of dimensions with IIC that was greater than 0.2 from the reference population was unsatisfactory in both populations. Moreover, the correlation for each item with its contributive dimension (IIC) was higher compared to the others (IDV) for 5 dimensions in the no/low- and highly-impaired populations. Cronbach’s alpha coefficients were satisfactory for 6 of the 8 dimensions in the no/low-impaired group. Furthermore, the INFIT statistics were satisfactory for both populations. The floor effects were less than 10% compared to the reference population for 6 dimensions in both groups, and the ceiling effects were less than 10% from the reference population for 4 and 5 dimensions in the no/low- and highly-impaired populations, respectively.

**Figure 1:**
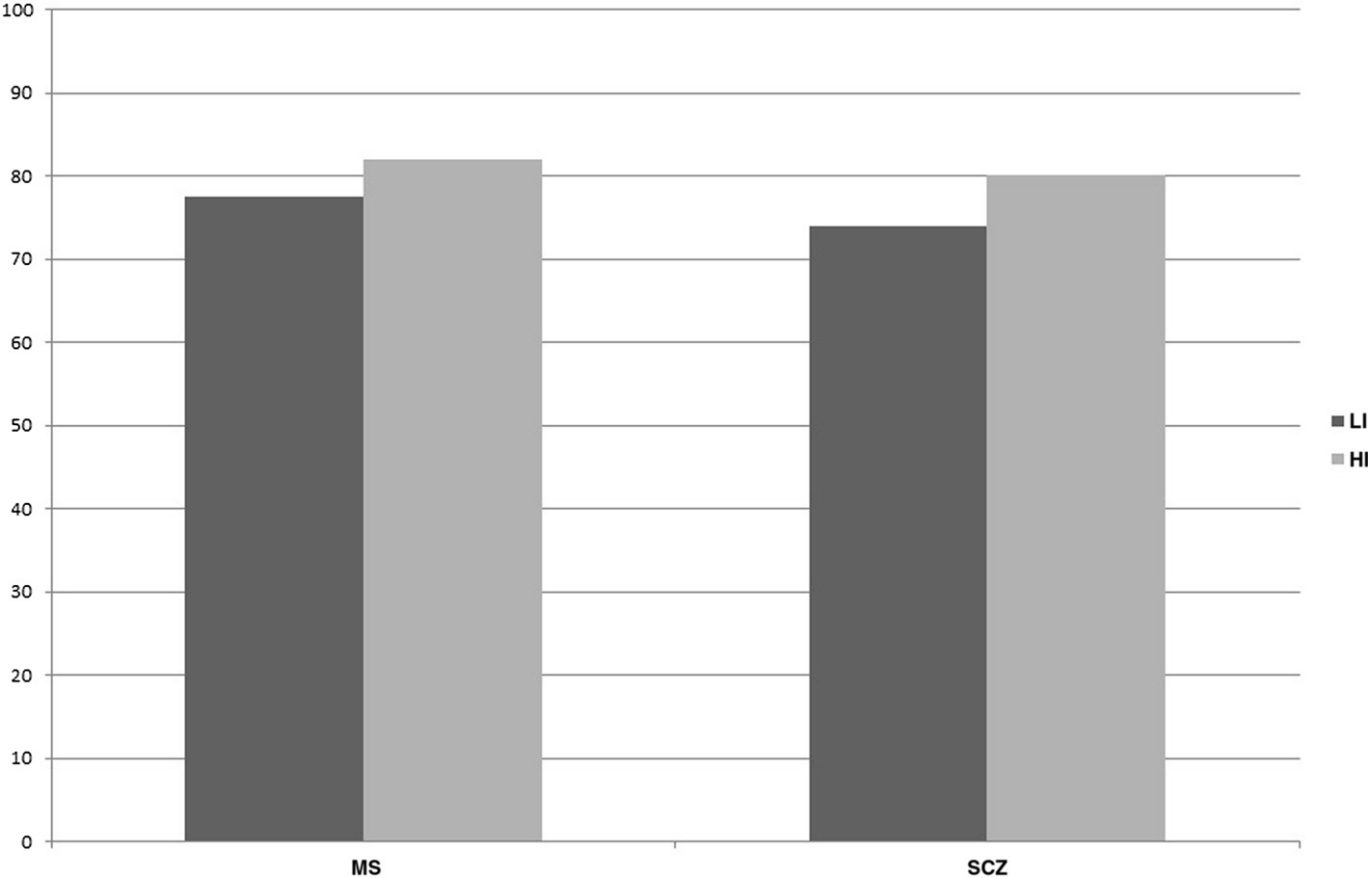
**Suitability indices of construct validity according to the disease.** LI no/low-impaired (0 or 1 impaired composites: executive, memory, and attention functions); HI highly-impaired (2 or 3 impaired composites). MS multiple sclerosis, SCZ schizophrenia.

The suitability indices of the construct validity were 80% in the highly-impaired population and 74% in the no/low-impaired population (Figure 1). Further details are provided in Table 2 and Table 3.

**Table 2:** Internal structural validity, reliability, unidimensionality according to the cognitive status

	**IIC**^**1 **^**Min-Max**			**IDV**^**2 **^**Min-Max**			**Floor %**			**Ceiling %**			**Alpha**^**3**^			**INFIT**^**4**^		
**MS**	**LI 50**	**HI 49**	***Ref 1992***	**LI 50**	**HI 49**	***Ref 1992***	**LI 50**	**HI 49**	***Ref 1992***	**LI 50**	**HI 49**	***Ref 1992***	**LI 50**	**HI 49**	***Ref 1992***	**LI 50**	**HI 49**	***Ref 1992***
ADL	0.48-0.69	0.19-0.78	*0.66-0.81*	-0.32-0.40	-0.15-0.39	*0,02-0,49*	29.50	35.73	*1,3*	8.25	5.88	*4,6*	0.86	0.84	*0,92*	0.66-1.76	0.47-2.15	*0,86-1,2*
PWB	0.55-0.82	0.52-0.76	0,67-0,76	-0.16-0.44	-0.06-0.60	0,09-0,41	7.50	13.25	2,4	17.00	14.78	4,6	0.88	0.81	0,85	0.79-1.30	0.68-1.20	0,81-1,13
RFr	0.74-0.83	0.78-0.84	0,69-0,78	-0.23-0.42	-0.17-0.29	0,04-0,36	2.67	5.43	2,4	18.00	14.97	13	0.89	0.89	0,75	0.70-1.13	0.71-1.17	0,84-1,15
SPT	0.41-0.58	0.42-0.60	0,48-0,65	-0.09-0.49	-0.18-0.41	0,06-0,41	5.50	16.35	0,7	23.00	15.30	10,3	0.69	0.72	0,80	0.72-1.15	0.85-1.14	0,75-1,17
RFa	0.58-0.61	0.63-0.65	0,62-0,68	-0.35-0.47	-0.18-0.39	0,04-0,38	3.00	2.00	0,8	44.67	35.40	25,7	0.76	0.77	0,86	0.96-1.05	0.92-1.08	0,88-1,07
RHCS	0.44-0.65	0.47-0.60	0,42-0,56	-0.18-0.34	-0.19-0.22	0,05-0,32	0.67	2.73	0,3	28.67	19.7	24,5	0.71	0.72	0,68	0.97-1.29	0.79-1.21	0,83-1,18
SSL	0.43	0.77	*0,75-0,75*	-0.13-0.48	-0.9-0.31	*0,15-0,43*	17.00	26.5	*7,6*	23.00	10.2	*18,7*	0.64	0.85	*0,85*	0.93-1.04	0.95-1.03	*0,98-1*
COP	0.29	0.61	*0,66-0,66*	-0.14-0.46	-0.17-0.39	*0,12-0,45*	11.00	16.3	*5,8*	29.00	19.35	*21,1*	0.43	0.75	*0,80*	0.99	0.94-0.97	*0,97-1*
REJ	0.77	0.76	*0,60-0,60*	-0.14-0.37	-0.17-0.55	*0,13-0,41*	3.00	11.2	*1,5*	37.00	44.9	*32,9*	0.87	0.88	*0,74*	1.00-1.01	0.91-0.97	*0,95-1,04*
**SCZ**	**LI 40**	**HI 67**	***Ref 507***	**LI 40**	**HI 67**	***Ref 507***	**LI 40**	**HI 67**	***Ref 507***	**LI 40**	**HI 67**	***Ref 507***	**LI 40**	**HI 67**	***Ref 507***	**LI 40**	**HI 67**	***Ref 507***
PsW	0,53 -0,63	0,4 -0,47	*0.80-0.81*	-0,15 -0,67	0,03 -0,44	*0.13-0.46*	0	3,3	*13.9*	27	18	*35.1*	0,76	0,63	*0.73*	0,89 -1,17	0,98 -1,08	*0.91-1.05*
SE	0,74 -0,74	0,68 -0,68	*0.89-0.90*	0,09 -0,77	0,09 -0,54	*0.17-0.55*	10,8	0	*12.3*	10,8	24,6	*22.2*	0,85	0,81	*0.74*	0,98 -1,01	0,97 -0,99	*0.98-0.99*
RFa	0,79 -0,79	0,77 -0,77	*0.91-0.92*	-0,28 -0,28	-0,07 -0,25	*0.06-0.27*	5,4	14,8	*12.8*	16,2	9,8	*24.2*	0,88	0,87	*0.81*	0,89 -0,9	0,97 -0,98	*0.96-1.02*
RFr	0,67 -0,67	0,68 -0,68	*0.88-0.89*	-0,03 -0,35	0,04 -0,53	*0.16-0.34*	8,1	16,4	*21.3*	2,7	13,1	*12.8*	0,8	0,81	*0.73*	0,92 -1,02	0,97 -1	*0.98-1.00*
Re	0,59 -0,76	0,47 -0,64	*0.80-0.83*	-0,01 -0,59	0,12 -0,51	*0.03-0.49*	0	0	*10.5*	16,2	21,3	*24.2*	0,82	0,73	*0.74*	0,74 -1,19	0,81 -1,2	*0.96-1.03*
PhW	0,44 -0,44	0,76 -0,76	*0.83-0.84*	-0,15 -0,67	0,12 -0,61	*0.13-0.51*	2,7	8,2	*13.3*	13,5	23	*16.0*	0,61	0,86	*0.79*	0,95 -0,99	0,95 -0,98	*0.97-0.99*
Au	0,72 -0,72	0,79 -0,79	*0.92-0.93*	-0,15 -0,68	-0,07 -0,57	*0.07-0.45*	2,7	4,9	*10.2*	16,2	13,1	*18.7*	0,84	0,88	*0.84*	0,94 -0,95	0,95 -0,99	*0.98-0.99*
SL	0,78 -0,78	0,49 -0,49	*0.88-0.89*	-0,01 -0,58	0,14 -0,63	*0.16-0.48*	18,9	19,7	*28.8*	10,8	3,3	*12.7*	0,88	0,65	*0.72*	0,93 -0,94	0,98 -1,04	*0.98-1.01*

^1^Item-Internal Consistency, ^2^Item Discriminant Validity, ^3^Cronbach’s alpha, ^4^Rasch statistics.

MS multiple sclerosis, SCZ schizophrenia.

MusiQoL: ADL activity of daily living, PWB psychological well-being, RFr relationships with friends, SPT symptoms, RFa relationships with family, RHCS relationships with health care system, SSL sentimental and sexual life, COP coping, REJ rejection.

SQoL: PsW psychological well-being, SE self-esteem, RFa family relationships, RFr relationships with friends, RE: resilience, PhW physical well-being, AU autonomy, SL sentimental life.

LI no/low-impaired (0 or 1 of the 3 composites is impaired: executive, memory, and attention functions).

HI highly-impaired (2 or 3 composites are impaired).

*Ref 1992* reference population of MusiQoL [30].

*Ref 507* reference population of SQoL [32].

Bold values: unsatisfactory values.

Italic characters: reference population values.

**Table 3:** Suitability indices of construct validity and external validity according to the cognitive status: a global approach

	**MS**		**SCZ**	
**Construct validity**	**LI 50**	**HI 49**	**LI 40**	**HI 67**
% of identified factors	77.8	88.9	100	100
% of well-projected items	87.1	93.6	88.9	88.9
% of dimensions with IIC non exceeded 0.2 from ref	77.8	94.4	37.5	37.5
% of dimensions with IDV non exceeded 0.2 from ref	55.6	61.1	37.5	100
% of dimensions with IDV < IIC	66.7	77.8	62.5	62.5
% of dimensions with Cronbach’s alpha coefficients > =0.7 or > =ref	66.7	100	87.5	75.0
% of dimensions with INFIT ranged [0.7-1.3]	88.9	83.3	100	100
% of dimensions with MV < 10% from ref	100	100	100	100
% of dimensions with Floor < 10% from ref	88.9	44.4	75.0	75.0
% of dimensions with Ceiling < 10% from ref	66.7	77.8	50.0	62.5
**External validity**	LI 50	HI 49	LI 40	HI 67
Age: % of dimensions with correlation coefficient < 0.40	100	100	100	100
Disease severity: % of dimensions meeting conditions*	100	88.9	75.0	100
Disease duration: % of dimensions with correlation coefficient < 0.40	100	100	100	100
Gender: % of dimensions with ES < 0.2 from ref	55.6	88.9	75.0	62.5
Educational level: % of dimensions with ES < 0.2 from ref	44.4	55.6	75.0	75.0

MS multiple sclerosis, SCZ schizophrenia.

*MusiQoL and EDSS, the two conditions were: i) correlation coefficient between ADL and EDSS > 0.4 and stronger than the other correlations for MusiQoL; ii) all other correlation coefficients inferior to 0.40. SQoL and PANSS, the condition was correlation coefficient < 0.30 or not statistically significant. The score was 100% when all the dimensions met the condition.

LI no/low-impaired (0 or 1 impaired composites: executive. memory. and attention functions). HI highly-impaired (2 or 3 composites are impaired).

### External validity

In MS individuals, women generally reported lower scores compared to men excepted in the sentimental and sexual life independent of cognitive status. The suitability index was more satisfactory in the highly-impaired population compared to the no- or low-impaired population. The proportion of dimensions with an effect size of less than 0.2 compared to the reference population for educational level was moderately satisfactory in both populations. In SCZ individuals, the proportion of dimensions with an effect size of less than 0.2 compared to the reference population for gender and occupational status was more satisfactory in both the no/low and highly-impaired groups.

As expected, age and disease duration almost never correlated with the QoL dimensions in the MS and SCZ populations. Findings related to relationships with disease severity and QoL scores were close compared to those of the 2 reference populations.

**Figure 2:**
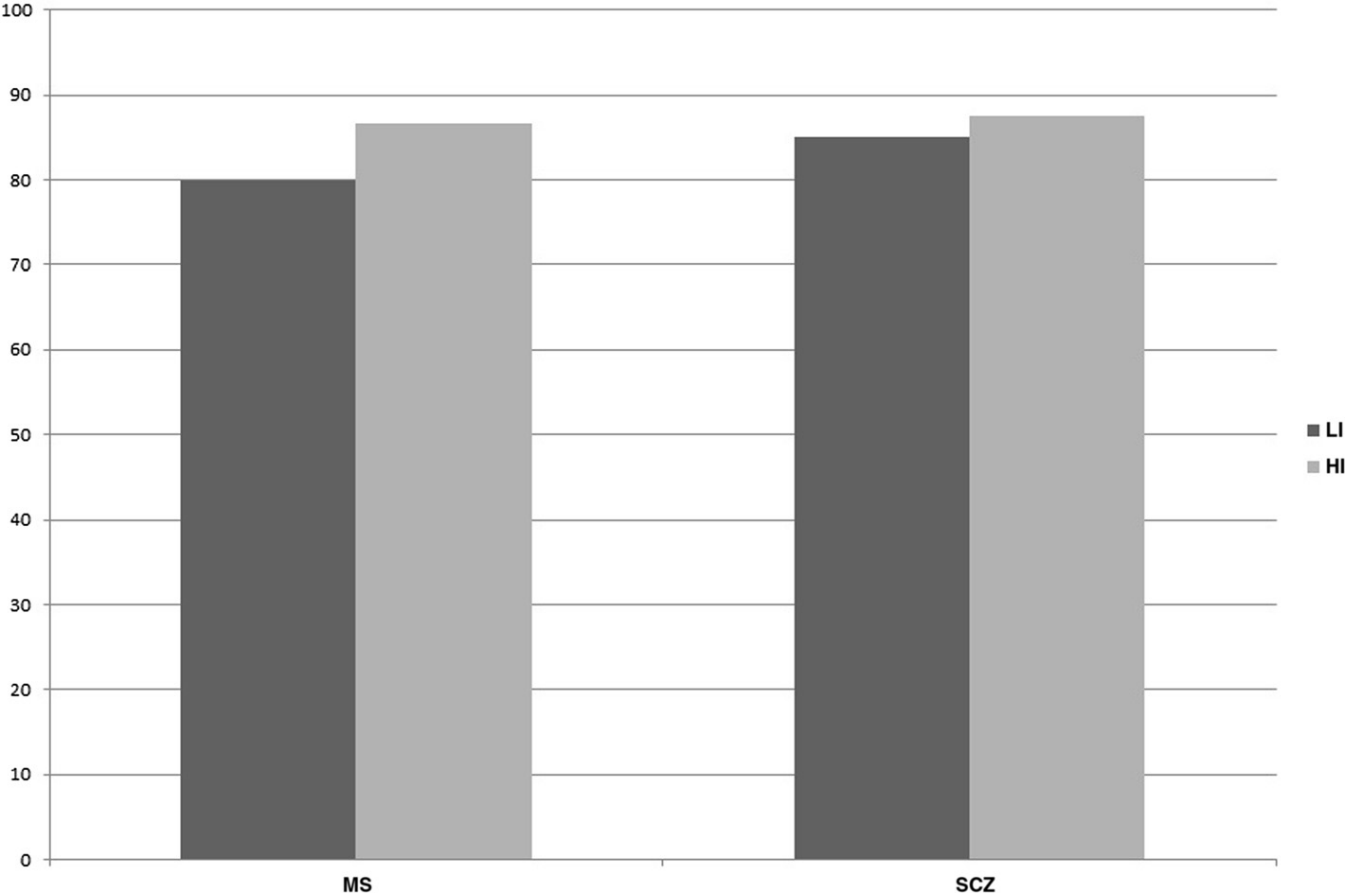
**Suitability indices of external validity according to the disease.** LI no/low-impaired (0 or 1 impaired composites: executive, memory, and attention functions); HI highly-impaired (2 or 3 impaired composites). MS multiple sclerosis, SCZ schizophrenia.

Taken together, the suitability indices of external validity in the no/low- and highly-impaired populations were 80% and 87% for the MS population and 85% and 88% for the SCZ population, respectively (Figure 2). These results are summarized in Tables 3, 4, and 5.

**Table 4:** Associations between QoL dimension scores and sociodemographic characteristics according to the cognitive status

**1. MS**		**Gender**			**Educational level**			**2. SCZ**		**Gender**			**Educational level**		
**MusiQoL**		**Women**	**Men**	**p**	**Low**	**High**	**p**	**SQoL**		**Women**	**Men**	**p**	**Low**	**High**	**p**
ADL	LI	32.9 ± 22.2	36.8 ± 20.9	0.29	22.4 ± 10.1	38.2 ± 22.6	**0.03**	PsW	LI	73.8 ± 31.3	75.3 ± 24.1	0.75	77.5 ± 20.4	73.2 ± 28.3	0.87
	HI	24.0 ± 16.3	29.9 ± 22.6	0.38	26.8 ± 16.6	25.7 ± 21.3	0.51		HI	56.3 ± 25.7	62.4 ± 27.1	0.30	56.5 ± 27.2	65.9 ± 24.9	0.08
PWB	LI	46.5 ± 27.4	62.0 ± 20.2	**0.03**	33.7 ± 21.3	59.3 ± 23.7	**0.00**	SE	LI	58.9 ± 33.6	62.9 ± 31.3	0.68	60.9 ± 33.5	63.0 ± 30.5	0.77
	HI	43.3 ± 25.2	57.8 ± 21.7	0.08	54.7 ± 27.0	42.8 ± 21.2	0.09		HI	59.5 ± 28.5	64.0 ± 30.8	0.53	62.2 ± 31.1	62.5 ± 28.3	1
RFr	LI	60.8 ± 25.5	64.5 ± 22.6	0.61	68.9 ± 26.1	60.7 ± 23.5	0.27	RFa	LI	75.0 ± 12.5	67.6 ± 29.1	0.96	63.3 ± 30.1	72.8 ± 24.3	0.36
	HI	66.1 ± 23.0	53.7 ± 25.8	0.06	58.0 ± 27.0	65.0 ± 22.0	0.36		HI	59.5 ± 37.2	64.6 ± 32.9	0.68	61.3 ± 36.6	65.0 ± 31.0	0.91
SPT	LI	55.6 ± 23.0	68.2 ± 18.0	**0.04**	47.7 ± 25.0	65.2 ± 19.1	**0.02**	RFr	LI	60.7 ± 19.7	47.7 ± 30.2	0.34	50.0 ± 30.9	50.0 ± 28.0	0.91
	HI	48.3 ± 25.5	50.7 ± 21.7	0.69	46.1 ± 22.4	52.3 ± 21.3	0.53		HI	37.5 ± 30.8	59.4 ± 32.1	**0.01**	49.7 ± 36.0	53.0 ± 28.7	0.88
RFa	LI	76.9 ± 21.1	75.0 ± 24.0	0.92	78.8 ± 27.0	75.2 ± 21.1	0.45	Re	LI	72.6 ± 22.9	67.7 ± 35.3	0.58	71.4 ± 26.3	66.7 ± 23.8	0.51
	HI	73.9 ± 23.2	70.4 ± 21.6	0.54	74.0 ± 23.7	71.1 ± 21.3	0.55		HI	57.3 ± 27.8	68.2 ± 25.5	0.10	62.1 ± 25.5	67.3 ± 38.7	0.32
RHCS	LI	73.5 ± 16.8	73.9 ± 17.5	0.69	85.6 ± 14.9	70.3 ± 16.1	**0.01**	PhW	LI	66.1 ± 25.7	64.8 ± 25.1	0.90	73.4 ± 21.3	59.2 ± 25.9	0.10
	HI	64.4 ± 16.4	65.7 ± 24.9	0.72	69.1 ± 20.3	60.8 ± 18.6	0.11		HI	45.8 ± 30.7	61.9 ± 32.8	**0.05**	52.5 ± 34.5	61.5 ± 29.8	0.30
SSL	LI	59.2 ± 30.0	50.0 ± 30.9	0.35	50.0 ± 33.9	56.1 ± 29.8	0.68	Au	LI	67.9 ± 34.5	68.4 ± 21.1	0.68	68.0 ± 22.8	68.5 ± 24.4	0.90
	HI	43.3 ± 31.6	33.3 ± 30.5	0.28	44.6 ± 36.3	35.2 ± 24.9	0.38		HI	59.0 ± 35.5	68.2 ± 24.7	0.41	64.6 ± 29.2	64.9 ± 30.0	0.94
COP	LI	53.7 ± 30.0	66.3 ± 21.1	0.07	46.6 ± 25.1	63.1 ± 26.4	0.09	SL	LI	58.3 ± 30.3	37.9 ± 32.1	0.15	43.4 ± 30.3	39.2 ± 34.4	0.62
	HI	47.1 ± 30.0	54.2 ± 32.9	0.45	49.0 ± 30.4	50.5 ± 32.2	0.89		HI	42.0 ± 31.0	45.5 ± 34.1	0.71	44.5 ± 33.4	43.8 ± 32.5	0.89
REJ	LI	59.3 ± 31.1	75.0 ± 25.6	0.06	58.0 ± 31.8	68.9 ± 28.8	0.29	Index	LI	66.5 ± 24.9	61.4 ± 16.0	0.36	63.5 ± 16.2	61.4 ± 18.5	0.80
	HI	63.8 ± 36.0	77.1 ± 30.4	0.18	68.8 ± 36.7	68.8 ± 32.6	0.67		HI	53.0 ± 19.6	61.4 ± 19.8	0.11	57.5 ± 18.8	59.3 ± 22.3	0.87
Index	LI	57.3 ± 11.4	63.3 ± 13.3	0.13	53.3 ± 15.7	62.2 ± 11.0	0.12								
	HI	52.7 ± 11.8	55.6 ± 15.5	0.36	54.0 ± 12.7	53.3 ± 13.7	0.86								

MS multiple sclerosis. SCZ schizophrenia.

MusiQoL: ADL activity of daily living. PWB psychological well-being. RFr relationships with friends. SPT symptoms. RFa relationships with family. RHCS relationships with health care system. SSL sentimental and sexual life. COP coping. REJ rejection.

SQoL: PsW psychological well-being. SE self-esteem. RFa family relationships. RFr relationships with friends. RE: resilience. PhW physical well-being. AU autonomy. SL sentimental life.

LI no/low-impaired (0 or 1 impaired composites: executive. memory. and attention functions). HI highly-impaired (2 or 3 composites are impaired).

Bold values: p < 0.05.

**Table 5:** Correlations between QoL dimension scores and age and clinical features according to the cognitive status

		**1. MS**					**2. SCZ**		
**MusiQoL**		**Age**	**EDSS**	**MS duration**	**SQoL**		**Age**	**PANSS**	**SCZ duration**
ADL	LI	-0.08	-0.46**	-0.01	PsW	LI	0.21	-0.34*	0.15
	HI	-0.05	-0.33*	-0.05		HI	0.08	-0.25*	0.16
PWB	LI	0.17	0.07	-0.07	SE	LI	0.05	-0.26	-0.08
	HI	0.11	0.08	-0.11		HI	0.04	0.00	0.10
RFr	LI	0.05	0.22	0.06	RFa	LI	-0.22	-0.12	-0.29
	HI	0.10	0.22	0.25		HI	-0.08	-0.19	0.07
SPT	LI	0.02	0.07	0.08	RFr	LI	-0.23	-0.18	-0.02
	HI	0.14	0.16	0.04		HI	-0.14	-0.08	0.08
RFa	LI	-0.06	-0.04	-0.27	Re	LI	0.13	-0.49**	0.10
	HI	-0.06	0.15	0.06		HI	-0.23	-0.14	-0.12
RHCS	LI	0.00	-0.09	-0.06	PhW	LI	0.05	-0.23	-0.03
	HI	0.16	-0.03	0.04		HI	-0.04	0.02	-0.05
SSL	LI	0.09	-0.02	0.10	Au	LI	0.32	-0.18	0.28
	HI	-0.19	-0.08	-0.17		HI	-0.02	0.00	-0.05
COP	LI	0.29*	0.07	0.11	SL	LI	0.13	-0.20	0.22
	HI	0.03	-0.03	-0.08		HI	0.17	-0.24	0.22
REJ	LI	0.28	0.13	0.09	Index	LI	0.01	-0.37*	0.04
	HI	0.00	0.20	0.06		HI	-0.08	-0.15	0.07
Index	LI	0.30*	0.08	0.16					
	HI	-0.01	0.09	-0.02					

MS multiple sclerosis. SCZ schizophrenia.

EDSS Expanded Disability Status Scale, PANSS Positive and Negative Syndrome Scale.

MusiQoL: ADL activity of daily living. PWB psychological well-being. RFr relationships with friends. SPT symptoms. RFa relationships with family. RHCS relationships with health care system. SSL sentimental and sexual life. COP coping. REJ rejection.

SQoL: PsW psychological well-being. SE self-esteem. RFa family relationships. RFr relationships with friends. RE: resilience. PhW physical well-being. AU autonomy. SL sentimental life.

LI no/low-impaired (0 or 1 impaired composites: executive. memory. and attention functions). HI highly-impaired (2 or 3 composites are impaired).

Spearman rank correlation coefficients were presented. *p-value <0.05. **p-value <0.01.

Bold values: p < 0.05.

## Discussion

Our results provide strong evidence supporting the relevance of self-reported quality of life assessments for patients with cognitive disorders, particularly in patients with severe cognitive dysfunction. It seems that the nature of multiple sclerosis and schizophrenia did not affect this type of assessment.

We examined these two diseases on the basis of the following points: i) the status of chronic illnesses with a high occurrence of reported cognitive deficits, even during the early disease stages [41,42]; ii) three main composites of cognition were indiscriminately affected [42,43]; iii) the homogeneous and extensive assessment of cognition, including tests assessing memory, attention, and executive function; iv) the availability of a disease-specific self-reported QoL questionnaire [30,32]; and v) the surprising similarities related to changes in white matter structure or abnormalities in myelin [24,25]. Moreover, some studies have suggested that changes in the integrity of white matter can result in impaired cognitive function in MS [44] and SCZ [45] patients.

These findings may support for the use of QoL assessment for clinicians who are still perplexed when interpreting the meaning of QoL scores for an individual with cognitive impairment. This present study suggests that cognitively impaired patients, as defined by a global cognitive dysfunction, can reliably and consistently respond to a specific QoL self-reported questionnaire. This assumption is underlined by the suitability indices found in the highly-impaired groups, i.e., 2 or 3 altered functions altered, in both MS and SCZ patients. These indices may be considered satisfactory compared to their respective reference populations. In the highly-impaired groups, factor analysis showed that the structure corresponded with the initial structure of the QoL questionnaires: 8 of the 9 dimensions were clearly identified in the MusiQoL and all the dimensions were identified in the SQoL. Although the IIC values reported in the highly-impaired population of MS individuals were similar to those identified in the reference population, the proportion of dimensions with IIC that exceeded 0.2 compared to the reference population was less satisfactory in SCZ individuals. For MS and SCZ populations, the proportion of dimensions with IDV values greater than the IIC values and the proportion of dimensions with IDV exceeding 0.2 compared to the reference population were less satisfactory, which may be explained by the very restricted definition of the decision rule. Internal consistency coefficients calculated in the highly-impaired groups were close to those of the initial reference populations. The floor and ceiling effects were slightly different compared to those reported in the initial validation publication independent of disease type. In addition, satisfactory INFIT statistics supported the unidimensionality of each of the dimensions.

Regarding external validity, highly-impaired populations showed satisfactory suitability indices. The links between QoL scores and age, severity disease score (EDSS and PANSS), and disease duration were closer to the initial reference populations independent of cognitive status and disease. However, links between QoL scores and gender and educational level were less satisfactory. In summary, the suitability indices of the highly-impaired population may be considered completely acceptable considering the small sample size of the defined populations.

Several previous studies have employed similar approaches to define cognitive dysfunction using a single composite, such as memory [7,15], attention [16], and executive functions [17,46]. It should be acknowledged that a single test of cognitive functioning would never be entirely appropriate to define an impaired cognitive population. One composite cannot be a perfect reflection of global cognitive function because patients suffer from several neuropsychological deficits. It would be unusual to observe one deficit in isolation [20,47-49], and QoL measurement may be altered depending on the type of cognitive impairment [50]. Thus, it is necessary to report additional information according to other definitions of cognitive dysfunction using a combination of different composites. To the best of our knowledge, this is the first study that uses the definition of cognitive dysfunction, which integrates a combination of different composites (i.e., memory, attention, and concentration).

Several limitations and strengths of this study should be mentioned:

1. The representativeness of the samples should be discussed. Compared to international and European cohorts, the MS patients in this study exhibited a more severe disability profile [30,51], and the SCZ patients had a longer illness duration [52]. Thus, an assessment of the reproducibility of our results is needed, using a larger and more diverse group of patients. However, the proportion of cognitively impaired subjects was consistent with the literature for MS [13,20,53] and SCZ [54,55] domains.

2. One important aspect of this study concerns our definition of cognitive dysfunction because there is little consensus according to Achiron and Barak [56]. We defined cognitive impairment using tests in which the French norms have been previously published [35-37]. This eliminated the need for a control group and enabled a consensus in defining patients as non-impaired or impaired for each test. However, our definition may be questionable because of the absence of a consensus on a ‘global definition’ of a patient with global cognitive dysfunction on the basis of a combination of these tests. However, we are convinced that our findings, independent of the definition of cognitive dysfunction, can help researchers to better understand the relevance of self-reported quality of life assessments for patients with cognitive disorders.

3. The suitability indices used to define the satisfactory properties relied on arbitrary decision rules, each of which will be discussed. Nevertheless, this approach enabled the determination of the suitability or unsuitability of different structures using the same decision tree independent of the questionnaire and disease. Thus, future studies may be performed to test different decision trees and to discuss the implications of the subsequent results.

4. Factors previously associated to cognitive performance, such as depression and fatigue [57,58], and medications [59] were not considered. However, the aim of this study was to provide evidence supporting the conclusion that cognitively impaired patients reliably answer a self-reported QoL questionnaire regardless of the presence or absence of other factors that could have influenced their performance.

## Conclusion

These findings confirmed preliminary results, which suggest that cognitive decline, as defined using a global cognitive dysfunction, did not compromise the reliability or validity of self-reported health measures. This study should support the clinical relevance of QoL assessment, thereby enhancing the use of QoL measures in clinical practice for cognitively impaired patients.

## Competing interest

The authors declare that they have no competing interests.

## Authors’ contribution

Conception and design: CL, JP, PA. Study coordination: KB, LB, CL, JP, PA. Inclusion and clinical data collection: CL, JP. Acquisition of cognitive data: VA, NP, FR. Analysis of data: KB, MB, AL. Interpretation of data: KB, MB, LB, VA, CL, JP, PA. Drafting and writing of manuscript: KB, PA. All the authors approved the manuscript.

## Pre-publication history

The pre-publication history for this paper can be accessed here:

http://www.biomedcentral.com/1471-2377/14/78/prepub

## References

[B1] Apolone G De Carli G Brunetti M Garattini S Health-related quality of life (HR-QOL) and regulatory issues. An assessment of the European Agency for the Evaluation of Medicinal Products (EMEA) recommendations on the use of HR-QOL measures in drug approval Pharmacoeconomics 2001 19 2 187 195 10.2165/00019053-200119020-00005 11284382

[B2] Bottomley A Jones D Claassens L Patient-reported outcomes: assessment and current perspectives of the guidelines of the Food and Drug Administration and the reflection paper of the European Medicines Agency Eur J Cancer 2009 45 3 347 353 10.1016/j.ejca.2008.09.032 19013787

[B3] Greenhalgh J Long AF Flynn R The use of patient reported outcome measures in routine clinical practice: lack of impact or lack of theory? Soc Sci Med 2005 60 4 833 843 10.1016/j.socscimed.2004.06.022 15571900

[B4] Awad AG Quality-of-life assessment in schizophrenia: the unfulfilled promise Expert Rev Pharmacoecon Outcomes Res 2011 11 5 491 493 10.1586/erp.11.61 21958091

[B5] Riemsma RP Forbes CA Glanville JM Eastwood AJ Kleijnen J General health status measures for people with cognitive impairment: learning disability and acquired brain injury Health Technol Assess 2001 5 6 1 100 10.3310/hta5060 11319989

[B6] Gold SM Schulz H Monch A Schulz KH Heesen C Cognitive impairment in multiple sclerosis does not affect reliability and validity of self-report health measures Mult Scler 2003 9 4 404 410 10.1191/1352458503ms927oa 12926847

[B7] Marrie RA Miller DM Chelune GJ Cohen JA Validity and reliability of the MSQLI in cognitively impaired patients with multiple sclerosis Mult Scler 2003 9 6 621 626 10.1191/1352458503ms971oa 14664477

[B8] Nishiyama T Ozaki N Measurement limit of quality-of-life questionnaires in psychiatric settings Qual Life Res 2010 19 1 25 30 10.1007/s11136-009-9556-1 19936965

[B9] Voruganti L Heslegrave R Awad AG Seeman MV Quality of life measurement in schizophrenia: reconciling the quest for subjectivity with the question of reliability Psychol Med 1998 28 1 165 172 10.1017/S0033291797005874 9483693

[B10] Baro E Ferrer M Vazquez O Miralles R Pont A Esperanza A Cervera AM Alonso J Using the Nottingham Health Profile (NHP) among older adult inpatients with varying cognitive function Qual Life Res 2006 15 4 575 585 10.1007/s11136-005-3691-0 16688491

[B11] Bureau-Chalot F Novella JL Jolly D Ankri J Guillemin F Blanchard F Feasibility, acceptability and internal consistency reliability of the nottingham health profile in dementia patients Gerontology 2002 48 4 220 225 10.1159/000058354 12053111

[B12] DePalma JA Measuring quality of life of patients of traumatic brain injury Crit Care Nurs Q 2001 23 4 42 51 10.1097/00002727-200102000-00004 11852949

[B13] Goverover Y Chiaravalloti N DeLuca J The relationship between self-awareness of neurobehavioral symptoms, cognitive functioning, and emotional symptoms in multiple sclerosis Mult Scler 2005 11 2 203 212 10.1191/1352458505ms1153oa 15794396

[B14] Benedict RH Cox D Thompson LL Foley F Weinstock-Guttman B Munschauer F Reliable screening for neuropsychological impairment in multiple sclerosis Mult Scler 2004 10 6 675 678 10.1191/1352458504ms1098oa 15584493

[B15] Baumstarck K Reuter F Boucekine M Aghababian V Klemina I Loundou A Pelletier J Auquier P Relevance of quality of life assessment for multiple sclerosis patients with memory impairment Plos ONE 2012 7 12 e50056 10.1371/journal.pone.0050056 23239975 PMC3519834

[B16] Baumstarck K Boucekine M Klemina I Reuter F Aghababian V Loundou A Pelletier J Auquier P What is the relevance of quality of life assessment for patients with attention impairment? Health Qual Life Outcomes 2013 11 70 10.1186/1477-7525-11-70 23618058 PMC3640938

[B17] Baumstarck K Boyer L Boucekine M Aghababian V Parola N Lancon C Auquier P Self-reported quality of life measure is reliable and valid in adult patients suffering from schizophrenia with executive impairment Schizophr Res 2013 147 1 58 67 10.1016/j.schres.2013.03.008 23566495

[B18] Juniper EF Guyatt GH Mesbah M Ravaud P Quality of life and pharmacoeconomics in clinical trials 1996 Philadelphia: Lippincott-Raven

[B19] Nunnaly JC Bernstein IC Psychometric theory 1994 New York: Mc Graw-Hill

[B20] Amato MP Zipoli V Portaccio E Multiple sclerosis-related cognitive changes: a review of cross-sectional and longitudinal studies J Neurol Sci 2006 245 1–2 41 46 16643953 10.1016/j.jns.2005.08.019

[B21] Feuillet L Reuter F Audoin B Malikova I Barrau K Cherif AA Pelletier J Early cognitive impairment in patients with clinically isolated syndrome suggestive of multiple sclerosis Mult Scler 2007 13 1 124 127 10.1177/1352458506071196 17294621

[B22] Elvevag B Goldberg TE Cognitive impairment in schizophrenia is the core of the disorder Crit Rev Neurobiol 2000 14 1 1 21 11253953

[B23] Green MF Kern RS Heaton RK Longitudinal studies of cognition and functional outcome in schizophrenia: implications for MATRICS Schizophr Res 2004 72 1 41 51 10.1016/j.schres.2004.09.009 15531406

[B24] Agartz I Andersson JL Skare S Abnormal brain white matter in schizophrenia: a diffusion tensor imaging study Neuroreport 2001 12 10 2251 2254 10.1097/00001756-200107200-00041 11447344

[B25] Bartzokis G Altshuler L Reduced intracortical myelination in schizophrenia Am J Psychiatry 2005 162 6 1229 1230 15930084 10.1176/appi.ajp.162.6.1229-a

[B26] Polman CH Reingold SC Banwell B Clanet M Cohen JA Filippi M Fujihara K Havrdova E Hutchinson M Kappos L Lublin FD Montalban X O'Connor P Sandberg-Wollheim M Thompson AJ Waubant E Weinshenker B Wolinsky JS Diagnostic criteria for multiple sclerosis: 2010 revisions to the McDonald criteria Ann Neurol 2011 69 2 292 302 10.1002/ana.22366 21387374 PMC3084507

[B27] APA DSM-IV. Diagnostic and Statistical Manual of Mental Disorders, 4th ed. Text revised 2000 Washington, DC: American Psychiatric Association

[B28] Kurtzke JF Rating neurologic impairment in multiple sclerosis: an expanded disability status scale (EDSS) Neurology 1983 33 11 1444 1452 10.1212/WNL.33.11.1444 6685237

[B29] Kay SR Opler LA Fiszbein A Significance of positive and negative syndromes in chronic schizophrenia Br J Psychiatry 1986 149 439 448 10.1192/bjp.149.4.439 3814927

[B30] Simeoni MC Auquier P Fernandez O Flachenecker P Stecchi S Constantinescu C Idiman E Boyko A Beiske A Vollmer T Triantafyllou N O'Connor P Barak Y Biermann L Cristiano E Atweh S Patrick D Robitail S Ammoury N Beresniak A Pelletier J Validation of the multiple sclerosis international quality of life questionnaire Mult Scler 2008 14 2 219 230 17942521 10.1177/1352458507080733

[B31] Auquier P Simeoni MC Sapin C Reine G Aghababian V Cramer J Lancon C Development and validation of a patient-based health-related quality of life questionnaire in schizophrenia: the S-QoL Schizophr Res 2003 63 1–2 137 149 12892868 10.1016/s0920-9964(02)00355-9

[B32] Boyer L Simeoni MC Loundou A D’Amato T Reine G Lancon C Auquier P The development of the S-QoL 18: a shortened quality of life questionnaire for patients with schizophrenia Schizophr Res 2011 121 1–3 241 250 20541912 10.1016/j.schres.2010.05.019

[B33] Jensen AR Scoring the stroop test Acta Psychol (Amst) 1965 24 5 398 408 5841721 10.1016/0001-6918(65)90024-7

[B34] Bowie CR Harvey PD Administration and interpretation of the trail making test Nat Protoc 2006 1 5 2277 2281 10.1038/nprot.2006.390 17406468

[B35] Van der Linden M Coyette F Poitrenaud J Kalafat M Calicis F Wyns C Adam S Solal L’épreuve de rappel libre, rappel indicé à 16 items L’évaluation des troubles de la mémoire Présentation de quatre tests de mémoire épisodique et étalonnage 2004 Marseille, France 25

[B36] Wechsler D Les Editions du Centre de Psychologie Appliquée WMS-R Echelle clinique de Mémoire de Wechsler-Révisée 1991 Paris, France

[B37] Godefroy Oet le Groupe de Reflexion pour l’Evaluation des Fonctions EXécutives (GREFEX). Fonctions exécutives et pathologies neurologiques et psychiatriques. Evaluation en pratique clinique, Solal edn 2008 Marseille, France: Collection Neuropsychologique

[B38] Ware JE Harris WJ Gandek B Rogers BW MAP-R for Windows: Multitrait-Multi-Item Analysis Program - Revised User’s Guide 1997 Boston: Health Assessment Lab

[B39] Cronbach LJ Coefficient alpha and the internal structure of tests Psychometrika 1951 16 297 334 10.1007/BF02310555

[B40] Wright BD Stone MH Best test design: Rasch measurement 1979 Chicago: Mesa press

[B41] Achiron A Barak Y Cognitive impairment in probable multiple sclerosis J Neurol Neurosurg Psychiatry 2003 74 4 443 446 10.1136/jnnp.74.4.443 12640060 PMC1738365

[B42] Braw Y Bloch Y Mendelovich S Ratzoni G Gal G Harari H Tripto A Levkovitz Y Cognition in young schizophrenia outpatients: comparison of first-episode with multiepisode patients Schizophr Bull 2008 34 3 544 554 17984299 10.1093/schbul/sbm115 PMC2632422

[B43] Zakzanis KK Distinct neurocognitive profiles in multiple sclerosis subtypes Arch Clin Neuropsychol 2000 15 2 115 136 10.1016/S0887-6177(98)00157-7 14590556

[B44] Kujala P Portin R Ruutiainen J The progress of cognitive decline in multiple sclerosis. A controlled 3-year follow-up Brain 1997 120 Pt 2 289 297 9117376 10.1093/brain/120.2.289

[B45] Fields RD White matter in learning, cognition and psychiatric disorders Trends Neurosci 2008 31 7 361 370 10.1016/j.tins.2008.04.001 18538868 PMC2486416

[B46] Baumstarck K Pelletier J Aghababian V Reuter F Klemina I Berbis J Loundou A Auquier P Is the concept of quality of life relevant for multiple sclerosis patients with cognitive impairment? Preliminary results of a cross-sectional study Plos ONE 2012 7 1 e30627 doi:30610.31371/journal.pone.0030627 10.1371/journal.pone.0030627 22292002 PMC3264575

[B47] Rao SM Leo GJ Bernardin L Unverzagt F Cognitive dysfunction in multiple sclerosis. I. Frequency, patterns, and prediction Neurology 1991 41 5 685 691 10.1212/WNL.41.5.685 2027484

[B48] Benedict RH Cookfair D Gavett R Gunther M Munschauer F Garg N Weinstock-Guttman B Validity of the minimal assessment of cognitive function in multiple sclerosis (MACFIMS) J Int Neuropsychol Soc 2006 12 4 549 558 16981607 10.1017/s1355617706060723

[B49] Chiaravalloti ND DeLuca J Cognitive impairment in multiple sclerosis Lancet Neurol 2008 7 12 1139 1151 10.1016/S1474-4422(08)70259-X 19007738

[B50] Benito-Leon J Morales JM Rivera-Navarro J Health-related quality of life and its relationship to cognitive and emotional functioning in multiple sclerosis patients Eur J Neurol 2002 9 5 497 502 10.1046/j.1468-1331.2002.00450.x 12220381

[B51] Amato MP Grimaud J Achiti I Bartolozzi ML Adeleine P Hartung HP Kappos L Thompson A Trojano M Vukusic S Confavreux C European validation of a standardized clinical description of multiple sclerosis J Neurol 2004 251 12 1472 1480 10.1007/s00415-004-0567-0 15645346

[B52] Bebbington PE Angermeyer M Azorin JM Brugha T Kilian R Johnson S Toumi M Kornfeld A The European Schizophrenia Cohort (EuroSC): a naturalistic prognostic and economic study Soc Psychiatry Psychiatr Epidemiol 2005 40 9 707 717 10.1007/s00127-005-0955-5 16151597

[B53] DeLuca J Barbieri-Berger S Johnson SK The nature of memory impairments in multiple sclerosis: acquisition versus retrieval J Clin Exp Neuropsychol 1994 16 2 183 189 10.1080/01688639408402629 8021305

[B54] Hughes C Kumari V Soni W Das M Binneman B Drozd S O’Neil S Mathew V Sharma T Longitudinal study of symptoms and cognitive function in chronic schizophrenia Schizophr Res 2003 59 2–3 137 146 12414070 10.1016/s0920-9964(01)00393-0

[B55] Dickinson D Ramsey ME Gold JM Overlooking the obvious: a meta-analytic comparison of digit symbol coding tasks and other cognitive measures in schizophrenia Arch Gen Psychiatry 2007 64 5 532 542 10.1001/archpsyc.64.5.532 17485605

[B56] Achiron A Barak Y Cognitive changes in early MS: a call for a common framework J Neurol Sci 2006 245 1–2 47 51 16635495 10.1016/j.jns.2005.05.019

[B57] Arnett PA Randolph JJ Longitudinal course of depression symptoms in multiple sclerosis J Neurol Neurosurg Psychiatry 2006 77 5 606 610 10.1136/jnnp.2004.047712 16614019 PMC2117462

[B58] Debouverie M Pittion-Vouyovitch S Brissart H Guillemin F Physical dimension of fatigue correlated with disability change over time in patients with multiple sclerosis J Neurol 2008 255 5 633 636 10.1007/s00415-008-0761-6 18217185

[B59] Oken BS Flegal K Zajdel D Kishiyama SS Lovera J Bagert B Bourdette DN Cognition and fatigue in multiple sclerosis: potential effects of medications with central nervous system activity J Rehabil Res Dev 2006 43 1 83 90 10.1682/JRRD.2004.11.0148 16847774

